# A Quick Introduction to Version Control with Git and GitHub

**DOI:** 10.1371/journal.pcbi.1004668

**Published:** 2016-01-19

**Authors:** John D. Blischak, Emily R. Davenport, Greg Wilson

**Affiliations:** 1 Committee on Genetics, Genomics, and Systems Biology, University of Chicago, Chicago, Illinois, United States of America; 2 Department of Molecular Biology and Genetics, Cornell University, Ithaca, New York, United States of America; 3 Software Carpentry Foundation, Toronto, Ontario, Canada; Ontario Institute for Cancer Research, CANADA

“This is part of the PLOS Computational Biology Education collection.”

## Introduction to Version Control

Many scientists write code as part of their research. Just as experiments are logged in laboratory notebooks, it is important to document the code you use for analysis. However, a few key problems can arise when iteratively developing code that make it difficult to document and track which code version was used to create each result. First, you often need to experiment with new ideas, such as adding new features to a script or increasing the speed of a slow step, but you do not want to risk breaking the currently working code. One often-utilized solution is to make a copy of the script before making new edits. However, this can quickly become a problem because it clutters your file system with uninformative filenames, e.g., analysis.sh, analysis_02.sh, analysis_03.sh, etc. It is difficult to remember the differences between the versions of the files and, more importantly, which version you used to produce specific results, especially if you return to the code months later. Second, you will likely share your code with multiple lab mates or collaborators, and they may have suggestions on how to improve it. If you email the code to multiple people, you will have to manually incorporate all the changes each of them sends.

Fortunately, software engineers have already developed software to manage these issues: version control. A version control system (VCS) allows you to track the iterative changes you make to your code. Thus, you can experiment with new ideas but always have the option to revert to a specific past version of the code you used to generate particular results. Furthermore, you can record messages as you save each successive version so that you (or anyone else) reviewing the development history of the code is able to understand the rationale for the given edits. It also facilitates collaboration. Using a VCS, your collaborators can make and save changes to the code, and you can automatically incorporate these changes to the main code base. The collaborative aspect is enhanced with the emergence of websites that host version-controlled code.

In this quick guide, we introduce you to one VCS, Git (https://git-scm.com), and one online hosting site, GitHub (https://github.com), both of which are currently popular among scientists and programmers in general. More importantly, we hope to convince you that although mastering a given VCS takes time, you can already achieve great benefits by getting started using a few simple commands. Furthermore, not only does using a VCS solve many common problems when writing code, it can also improve the scientific process. By tracking your code development with a VCS and hosting it online, you are performing science that is more transparent, reproducible, and open to collaboration [1,2]. There is no reason this framework needs to be limited only to code; a VCS is well-suited for tracking any plain-text files: manuscripts, electronic lab notebooks, protocols, etc.

## Version Your Code

The first step is to learn how to version your own code. In this tutorial, we will run Git from the command line of the Unix shell. Thus, we expect readers are already comfortable with navigating a filesystem and running basic commands in such an environment. You can find directions for installing Git for the operating system running on your computer by following one of the links provided in Table 1. There are many graphical user interfaces (GUIs) available for running Git (Table 1), which we encourage you to explore, but learning to use Git on the command line is necessary for performing more advanced operations and using Git on a remote machine.

**Table 1:** Resources.

Resource	Options
Distributed VCS	Git (https://git-scm.com)
	Mercurial (https://mercurial.selenic.com)
	Bazaar (http://bazaar.canonical.com)
Online hosting site	GitHub (https://github.com)
	Bitbucket (https://bitbucket.org)
	GitLab (https://about.gitlab.com)
	Source Forge (http://sourceforge.net)
Git installation	https://git-scm.com/downloads
Git tutorials	Software Carpentry (https://swcarpentry.github.io/git-novice)
	Pro Git (https://git-scm.com/book)
	A Visual Git Reference (https://marklodato.github.io/visual-git-guide)
	tryGit (https://try.github.io)
Graphical User Interface for Git	https://git-scm.com/downloads/guis

To follow along, first create a folder in your home directory named thesis. Next, download the three files provided in Supporting Information and place them in the thesis directory. Imagine that, as part of your thesis, you are studying the transcription factor CTCF, and you want to identify high-confidence binding sites in kidney epithelial cells. To do this, you will utilize publicly available ChIP-seq data produced by the ENCODE consortium [3]. ChIP-seq is a method for finding the sites in the genome where a transcription factor is bound, and these sites are referred to as peaks [4]. process.sh downloads the ENCODE CTCF ChIP-seq data from multiple types of kidney samples and calls peaks (S1 Data); clean.py filters peaks with a fold change cutoff and merges peaks from the different kidney samples (S2 Data); and analyze.R creates diagnostic plots on the length of the peaks and their distribution across the genome (S3 Data).

If you have just installed Git, the first thing you need to do is provide some information about yourself, since it records who makes each change to the file(s). Set your name and email by running the following lines, but replacing “First Last” and “user@domain” with your full name and email address, respectively.

$ git config --global user.name "First Last"

$ git config --global user.email "user@domain"

**Fig 1:**
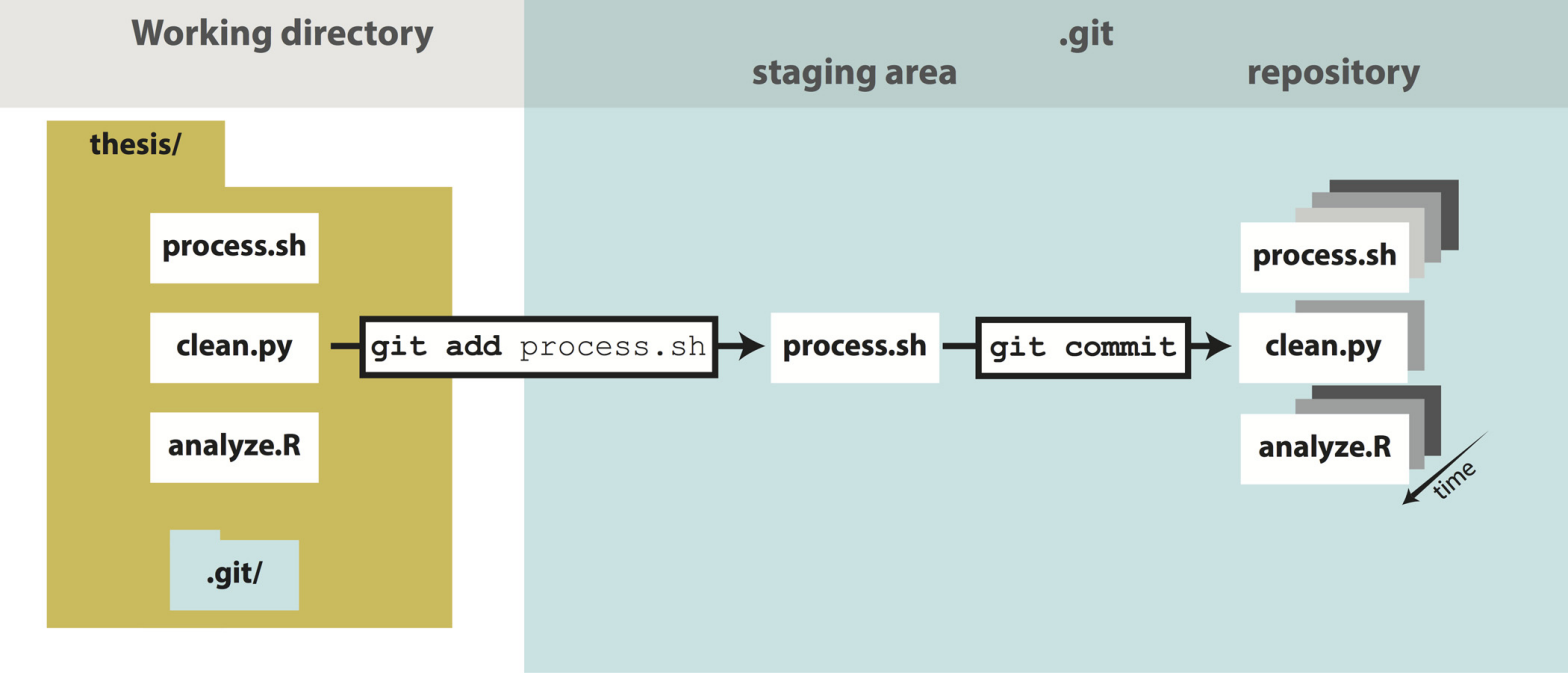
The git add/commit process. To store a snapshot of changes in your repository, first git add any files to the staging area you wish to commit (for example, you’ve updated the process.sh file). Second, type git commit with a message. Only files added to the staging area will be committed. All past commits are located in the hidden .git directory in your repository.

**Fig 2:**
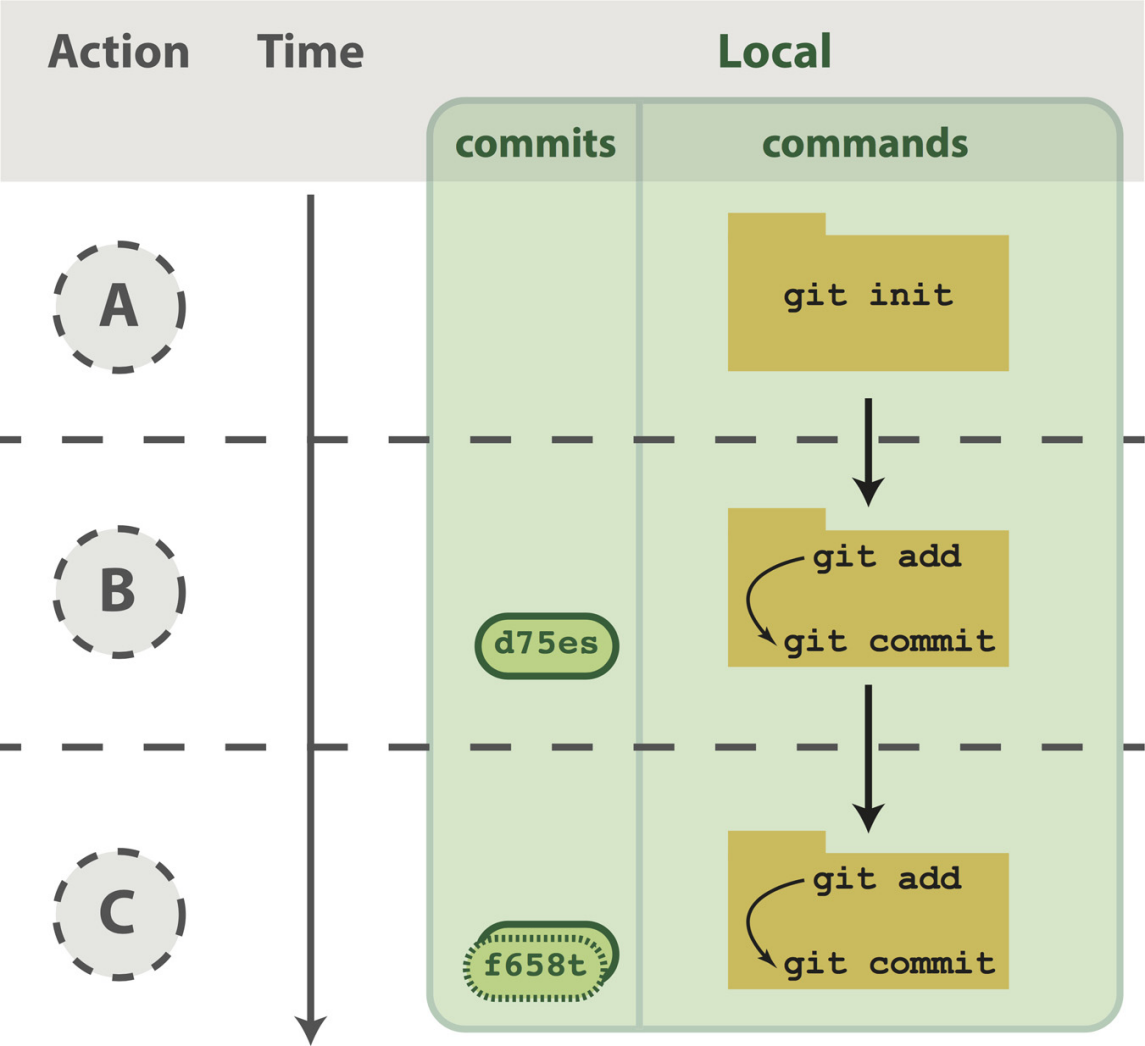
Working with a local repository. (A) To designate a directory on your computer as a Git repo, type the command git init. This initializes the repository and will allow you to track the files located within that directory. (B) Once you have added a file, follow the git add/commit cycle to place the new file first into the staging area by typing git add to designate it to be committed, and then git commit to take the shapshot of that file. The commit is assigned a commit identifier (d75es) that can be used in the future to pull up this version or to compare different committed versions of this file. (C) As you continue to add and change files, you should regularly add and commit those changes. Here, an additional commit was done, and the commit log now shows two commit identifiers: d75es (from step B) and f658t (the new commit). Each commit will generate a unique identifier, which can be examined in reverse chronological order using git log.

To start versioning your code with Git, navigate to your newly created directory, ~/thesis. Run the command git init to initialize the current folder as a Git repository (Figs 1 and 2A). A repository (or repo, for short) refers to the current version of the tracked files as well as all the previously saved versions (Box 1). Only files that are located within this directory (and any subdirectories) have the potential to be version controlled, i.e., Git ignores all files outside of the initialized directory. For this reason, projects under version control tend to be stored within a single directory to correspond with a single Git repository. For strategies on how to best organize your own projects, see Noble, 2009 [5].

$ cd ~/thesis

$ ls

analyze.R clean.py process.sh

$ git init

Initialized empty Git repository in ~/thesis/.git/

**Box 1. Definitions**

- **Version Control System (VCS)**: *(noun)* a program that tracks changes to specified files over time and maintains a library of all past versions of those files
- **Git**: *(noun)* a version control system
- **repository (repo)**: *(noun)* folder containing all tracked files as well as the version control history
- **commit**: *(noun)* a snapshot of changes made to the staged file(s); *(verb)* to save a snapshot of changes made to the staged file(s)
- **stage**: *(noun)* the staging area holds the files to be included in the next commit; *(verb)* to mark a file to be included in the next commit
- **track**: *(noun)* a tracked file is one that is recognized by the Git repository
- **branch**: *(noun)* a parallel version of the files in a repository (Box 7)
- **local**: *(noun)* the version of your repository that is stored on your personal computer
- **remote**: *(noun)* the version of your repository that is stored on a remote server; for instance, on GitHub
- **clone**: *(verb)* to create a local copy of a remote repository on your personal computer
- **fork**: *(noun)* a copy of another user’s repository on GitHub; *(verb)* to copy a repository; for instance, from one user’s GitHub account to your own
- **merge**: *(verb)* to update files by incorporating the changes introduced in new commits
- **pull**: *(verb)* to retrieve commits from a remote repository and merge them into a local repository
- **push**: *(verb)* to send commits from a local repository to a remote repository
- **pull request**: *(noun)* a message sent by one GitHub user to merge the commits in their remote repository into another user’s remote repository

Now you are ready to start versioning your code (Fig 1). Conceptually, Git saves snapshots of the changes you make to your files whenever you instruct it to. For instance, after you edit a script in your text editor, you save the updated script to your thesis folder. If you tell Git to save a shapshot of the updated document, then you will have a permanent record of the file in that exact version even if you make subsequent edits to the file. In the Git framework, any changes you have made to a script but have not yet recorded as a snapshot with Git reside in the working directory only (Fig 1). To follow what Git is doing as you record the initial version of your files, use the informative command git status.

$ git status

On branch master

Initial commit

Untracked files:

(use "git add <file>…" to include in what will be committed)

analyze.R

clean.py

process.sh

nothing added to commit but untracked files present (use "git add" to track)

There are a few key things to notice from this output. First, the three scripts are recognized as untracked files because you have not told Git to start tracking anything yet. Second, the word “commit” is Git terminology for a snapshot. As a noun, it means “a version of the code,” e.g., “the figure was generated using the commit from yesterday” (Box 1). This word can also be used as a verb, meaning “to save,” e.g., “to commit a change.” Lastly, the output explains how you can track your files using git add. Start tracking the file process.sh.

$ git add process.sh

And check its new status.

$ git status

On branch master

Initial commit

Changes to be committed:

(use "git rm --cached <file>…" to unstage)

new file: process.sh

Untracked files:

(use "git add <file>…" to include in what will be committed)

analyze.R

clean.py

Since this is the first time that you have told Git about the file process.sh, two key things have happened. First, this file is now being tracked, which means Git recognizes it as a file you wish to be version controlled (Box 1). Second, the changes made to the file (in this case the entire file, because it is the first commit) have been added to the staging area (Fig 1). Adding a file to the staging area will result in the changes to that file being included in the next commit, or snapshot, of the code (Box 1). As an analogy, adding files to the staging area is like putting things in a box to mail off, and committing is like putting the box in the mail.

Since this will be the first commit, or first version, of the code, use git add to begin tracking the other two files and add their changes to the staging area as well. Then create the first commit using the command git commit.

$ git add clean.py analyze.R

$ git commit -m "Add initial version of thesis code."

[master (root-commit) 660213b] Add initial version of thesis code.

3 files changed, 154 insertions(+)

create mode 100644 analyze.R

create mode 100644 clean.py

create mode 100644 process.sh

Notice the flag -m was used to pass a message for the commit. This message describes the changes that have been made to the code and is required. If you do not pass a message at the command line, the default text editor for your system will open so you can enter the message. You have just performed the typical development cycle with Git: make some changes, add updated files to the staging area, and commit the changes as a snapshot once you are satisfied with them (Fig 2).

Since Git records all of the commits, you can always look through the complete history of a project. To view the record of your commits, use the command git log. For each commit, it lists the unique identifier for that revision, author, date, and commit message.

$ git log

commit 660213b91af167d992885e45ab19f585f02d4661

Author: First Last <user@domain>

Date: Fri Aug 21 14:52:05 2015–0500

Add initial version of thesis code.

The commit identifier can be used to compare two different versions of a file, restore a file to a previous version from a past commit, and even retrieve tracked files if you accidentally delete them.

Now you are free to make changes to the files knowing that you can always revert them to the state of this commit by referencing its identifier. As an example, edit clean.py so that the fold change cutoff for filtering peaks is more stringent. Here is the current bottom of the file.

$ tail clean.py

# Filter based on fold-change over control sample

fc_cutoff = 10

epithelial = epithelial.filter(filter_fold_change, fc = fc_cutoff).saveas()

proximal_tube = proximal_tube.filter(filter_fold_change, fc = fc_cutoff).saveas()

kidney = kidney.filter(filter_fold_change, fc = fc_cutoff).saveas()

# Identify only those sites that are peaks in all three tissue types

combined = pybedtools.BedTool().multi_intersect(

i = [epithelial.fn, proximal_tube.fn, kidney.fn])

union = combined.filter(lambda x: int(x[3]) = = 3).saveas()

union.cut(range(3)).saveas(data + "/sites-union.bed")

Using a text editor, increase the fold change cutoff from 10 to 20.

$ tail clean.py

# Filter based on fold-change over control sample

fc_cutoff = 20

epithelial = epithelial.filter(filter_fold_change, fc = fc_cutoff).saveas()

proximal_tube = proximal_tube.filter(filter_fold_change, fc = fc_cutoff).saveas()

kidney = kidney.filter(filter_fold_change, fc = fc_cutoff).saveas()

# Identify only those sites that are peaks in all three tissue types

combined = pybedtools.BedTool().multi_intersect(

i = [epithelial.fn, proximal_tube.fn, kidney.fn])

union = combined.filter(lambda x: int(x[3]) = = 3).saveas()

union.cut(range(3)).saveas(data + "/sites-union.bed")

Because Git is tracking clean.py, it recognizes that the file has been changed since the last commit.

$ git status

# On branch master

# Changes not staged for commit:

#    (use "git add <file>…" to update what will be committed)

#    (use "git checkout --<file>…" to discard changes in working directory)

#

#    modified: clean.py

#

no changes added to commit (use "git add" and/or "git commit -a")

The report from git status indicates that the changes to clean.py are not staged, i.e., they are in the working directory (Fig 1). To view the unstaged changes, run the command git diff.

$ git diff

diff --git a/clean.py b/clean.py

index 7b8c058.76d84ce 100644

--- a/clean.py

+++ b/clean.py

@@ -28,7 +28,7 @@ def filter_fold_change(feature, fc = 1):

return False

# Filter based on fold-change over control sample

-fc_cutoff = 10

+fc_cutoff = 20

epithelial = epithelial.filter(filter_fold_change, fc = fc_cutoff).saveas()

proximal_tube = proximal_tube.filter(filter_fold_change, fc = fc_cutoff).saveas()

kidney = kidney.filter(filter_fold_change, fc = fc_cutoff).saveas()

Any lines of text that have been added to the script are indicated with a +, and any lines that have been removed with a -. Here, we altered the line of code that sets the value of fc_cutoff. git diff displays this change as the previous line being removed and a new line being added with our update incorporated. You can ignore the first five lines of output, because they are directions for other software programs that can merge changes to files. If you wanted to keep this edit, you could add clean.py to the staging area using git add and then commit the change using git commit, as you did above. Instead, this time undo the edit by following the directions from the output of git status to “discard changes in the working directory” using the command git checkout.

$ git checkout -- clean.py

$ git diff

Now git diff returns no output, because git checkout undid the unstaged edit you had made to clean.py. This ability to undo past edits to a file is not limited to unstaged changes in the working directory. If you had committed multiple changes to the file clean.py and then decided you wanted the original version from the initial commit, you could replace the argument -- with the commit identifier of the first commit you made above (your commit identifier will be different; use git log to find it). The -- used above was simply a placeholder for the first argument because, by default, git checkout restores the most recent version of the file from the staging area (if you haven’t staged any changes to this file, as is the case here, the version of the file in the staging area is identical to the version in the last commit). Instead of using the entire commit identifier, use only the first seven characters, which is simply a convention, since this is usually long enough for it to be unique.

$ git checkout 660213b clean.py

At this point, you have learned the commands needed to version your code with Git. Thus, you already have the benefits of being able to make edits to files without copying them first, to create a record of your changes with accompanying messages, and to revert to previous versions of the files if needed. Now you will always be able to recreate past results that were generated with previous versions of the code (see the command git tag for a method to facilitate finding specific past versions) and see the exact changes you have made over the course of a project.

## Share Your Code

**Fig 3:**
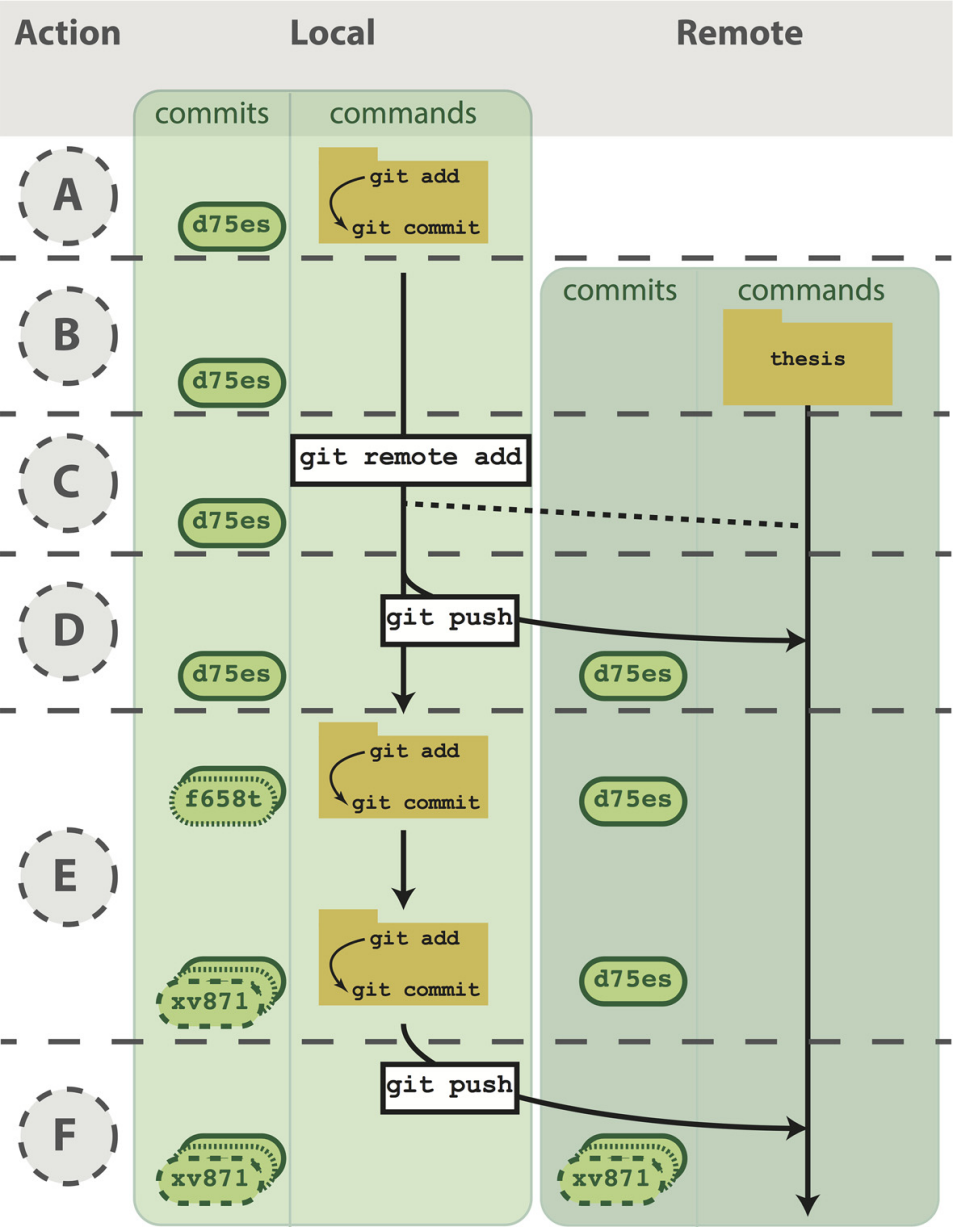
Working with both a local and remote repository as a single user. (A) On your computer, you commit to a Git repository (commit d75es). (B) On GitHub, you create a new repository called thesis. This repository is currently empty and not linked to the repo on your local machine. (C) The command git remote add connects your local repository to your remote repository. The remote repository is still empty, however, because you have not pushed any content to it. (D) You send all the local commits to the remote repository using the command git push. Only files that have been committed will appear in the remote repository. (E) You repeat several more rounds of updating scripts and committing on your local computer (commit f658t and then commit xv871). You have not yet pushed these commits to the remote repository, so only the previously pushed commit is in the remote repo (commit d75es). (F) To bring the remote repository up to date with your local repository, you git push the two new commits to the remote repository. The local and remote repositories now contain the same files and commit histories.

Once you have your files saved in a Git repository, you can share it with your collaborators and the wider scientific community by putting your code online (Fig 3). This also has the added benefit of creating a backup of your scripts and provides a mechanism for transferring your files across multiple computers. Sharing a repository is made easier if you use one of the many online services that host Git repositories (Table 1), e.g., GitHub. Note, however, that any files that have not been tracked with at least one commit are not included in the Git repository, even if they are located within the same directory on your local computer (see Box 2 for advice on the types of files that should not be versioned with Git and Box 3 for advice on managing large files).

**Box 2. What Not to Version Control**

You can version control any file that you put in a Git repository, whether it is text-based, an image, or a giant data file. However, just because you *can* version control something, does not mean you *should*. Git works best for plain, text-based documents such as your scripts or your manuscript if written in LaTeX or Markdown. This is because for text files, Git saves the entire file only the first time you commit it and then saves just your changes with each commit. This takes up very little space, and Git has the capability to compare between versions (using git diff). You can commit a non-text file, but a full copy of the file will be saved in each commit that modifies it. Over time, you may find the size of your repository growing very quickly. A good rule of thumb is to version control anything text-based: your scripts or manuscripts if they are written in plain text. Things *not* to version control are large data files that never change, binary files (including Word and Excel documents), and the output of your code.

In addition to the type of file, you need to consider the content of the file. If you plan on sharing your commits publicly using GitHub, ensure you are not committing any files that contain sensitive information, such as human subject data or passwords.

To prevent accidentally committing files you do not wish to track, and to remove them from the output of git status, you can create a file called .gitignore. In this file, you can list subdirectories and/or file patterns that Git should ignore. For example, if your code produced log files with the file extension .log, you could instruct Git to ignore these files by adding *.log to .gitignore. In order for these settings to be applied to all instances of the repository, e.g., if you clone it onto another computer, you need to add and commit this file.

**Box 3. Managing Large Files**

Many biological applications require handling large data files. While Git is best suited for collaboratively writing small text files, nonetheless, collaboratively working on projects in the biological sciences necessitates managing this data.

The example analysis pipeline in this tutorial starts by downloading data files in BAM format that contain the alignments of short reads from a ChIP-seq experiment to the human genome. Since these large, binary files are not going to change, there is no reason to version them with Git. Thus, hosting them on a remote http (as ENCODE has done in this case) or ftp site allows each collaborator to download it to her machine as needed, e.g., using wget, curl, or rsync. If the data files for your project are smaller, you could also share them via services like Dropbox (www.dropbox.com) or Google Drive (https://www.google.com/drive/).

However, some intermediate data files may change over time, and the practical necessity to ensure all collaborators are using the same data set may override the advice to *not* put code output under version control, as described in Box 2. Again, returning to the ChIP-seq example, the first step calling the peaks is the most difficult computationally because it requires access to a Unix-like environment and sufficient computational resources. Thus, for collaborators that want to experiment with clean.py and analyze.R without having to run process.sh, you could version the data files containing the ChIP-seq peaks (which are in BED format). But since these files are larger than those typically used with Git, you can instead use one of the solutions for versioning large files within a Git repository without actually saving the file with Git, e.g., git-annex (https://git-annex.branchable.com/) or git-fat (https://github.com/jedbrown/git-fat/). Recently, GitHub has created their own solution for managing large files called Git Large File Storage (LFS) (https://git-lfs.github.com/). Instead of committing the entire large file to Git, which quickly becomes unmanageable, it commits a text pointer. This text pointer refers to a specific file saved on a remote GitHub server. Thus, when you clone a repository, it only downloads the latest version of the large file. If you check out an older version of the repository, it automatically downloads the old version of the large file from the remote server. After installing Git LFS, you can manage all the BED files with one command: git lfs track "*.bed". Then you can commit the BED files just like your scripts, and they will automatically be handled with Git LFS. Now, if you were to change the parameters of the peak calling algorithm and re-run process.sh, you could commit the updated BED files, and your collaborators could pull the new versions of the files directly to their local Git repositories.

Below, we focus on the technical aspects of sharing your code. However, there are also other issues to consider when deciding if and how you are going to make your code available to others. For quick advice on these subjects, see Box 4 on how to license your code, Box 5 on concerns about being scooped, and Box 6 on the increasing trend of journals to institute sharing policies that require authors to deposit code in a public archive upon publication.

**Box 4. Choosing a License**

Putting software and other material in a public place is not the same as making it publicly usable. In order to do that, the authors must also add a license, since copyright laws in some jurisdictions require people to treat anything that isn’t explicitly open as being proprietary.

While dozens of open licenses have been created, the two most widely used are the GNU Public License (GPL) and the MIT/BSD family of licenses. Of these, the MIT/BSD-style licenses put the fewest requirements on re-use, and thereby make it easier for people to integrate your software into their projects.

For an excellent short discussion of these issues, and links to more information, see Jake Vanderplas’s blog post from March 2014 at http://www.astrobetter.com/blog/2014/03/10/the-whys-and-hows-of-licensing-scientific-code/. For a more in-depth discussion of the legal implications of different licenses, see Morin et al., 2012 [6].

**Box 5. Being Scooped**

One concern scientists frequently have about putting work in progress online is that they will be scooped, e.g., that someone will analyze their data and publish a result that they themselves would have, but hadn’t yet. In practice, though, this happens rarely, if at all: in fact, the authors are not aware of a single case in which this has actually happened, and would welcome pointers to specific instances. In practice, it seems more likely that making work public early in something like a version control repository, which automatically adds timestamps to content, will help researchers establish their priority.

**Box 6. Journal Policies**

Sharing data, code, and other materials is quickly moving from “desired” to “required.” For example, PLOS’s sharing policy (http://journals.plos.org/plosone/s/materials-and-software-sharing) already says, “We expect that all researchers submitting to PLOS will make all relevant materials that may be reasonably requested by others available without restrictions upon publication of the work.” Its policy on software is more specific:

We expect that all researchers submitting to PLOS submissions in which software is the central part of the manuscript will make all relevant software available without restrictions upon publication of the work. Authors must ensure that software remains usable over time regardless of versions or upgrades…

It then goes on to specify that software must be based on open source standards, and that it must be put in an archive which is large or long-lived. Granting agencies, philanthropic foundations, and other major sponsors of scientific research are all moving in the same direction, and, to our knowledge, none has relaxed or reduced sharing requirements in the last decade.

To begin using GitHub, you will first need to sign up for an account. For the code examples in this tutorial, you will need to replace username with the username of your account. Next, choose the option to “Create a new repository” (Fig 3B, see https://help.github.com/articles/create-a-repo/). Call it “thesis,” because that is the directory name containing the files on your computer, but note that you can give it a different name on GitHub if you wish. Also, now that the code will exist in multiple places, you need to learn some more terminology (Box 1). A local repository refers to code that is stored on the machine you are using, e.g., your laptop; whereas a remote repository refers to the code that is hosted online. Thus, you have just created a remote repository.

Now you need to send the code on your computer to GitHub. The key to this is the URL that GitHub assigns your newly created remote repository. It will have the form https://github.com/username/thesis.git (see https://help.github.com/articles/cloning-a-repository/). Notice that this URL is using the HTTPS protocol, which is the quickest to begin using. However, it requires you to enter your username and password when communicating with GitHub, so you’ll want to consider switching to the SSH protocol once you are regularly using Git and GitHub (see https://help.github.com/articles/generating-ssh-keys/ for directions). In order to link the local thesis repository on your computer to the remote repository you just created, in your local repository, you need to tell Git the URL of the remote repository using the command git remote add (Fig 3C).

$ git remote add origin https://github.com/username/thesis.git

The name “origin” is a bookmark for the remote repository so that you do not have to type out the full URL every time you transfer your changes (this is the default name for a remote repository, but you could use another name if you like).

Send your code to GitHub using the command git push (Fig 3D).

$ git push origin master

You first specify the remote repository, “origin.” Second, you tell Git to push to the “master” copy of the repository—we will not go into other options in this tutorial, but Box 7 discusses them briefly.

**Box 7. Branching**

Do you ever make changes to your code, but are not sure you will want to keep those changes for your final analysis? Or do you need to implement new features while still providing a stable version of the code for others to use? Using Git, you can maintain parallel versions of your code that you can easily bounce between while you are working on your changes. You can think of it like making a copy of the folder you keep your scripts in, so that you have your original scripts intact but also have the new folder where you make changes. Using Git, this is called branching, and it is better than separate folders because (1) it uses a fraction of the space on your computer, (2) it keeps a record of when you made the parallel copy (branch) and what you have done on the branch, and (3) there is a way to incorporate those changes back into your main code if you decide to keep your changes (and a way to deal with conflicts). By default, your repository will start with one branch, usually called “master.” To create a new branch in your repository, type git branch new_branch_name. You can see what branches a current repository has by typing git branch, with the branch you are currently in being marked by a star. To move between branches, type git checkout branch_to_move_to. You can edit files and commit them on each branch separately. If you want to combine the changes in your new branch with the master branch, you can merge the branches by typing git merge new_branch_name while in the master branch.

Pushing to GitHub also has the added benefit of backing up your code in case anything were to happen to your computer. Also, it can be used to manually transfer your code across multiple machines, similar to a service like Dropbox (www.dropbox.com) but with the added capabilities and control of Git. For example, what if you wanted to work on your code on your computer at home? You can download the Git repository using the command git clone.

$ git clone https://github.com/username/thesis.git

By default, this will download the Git repository into a local directory named “thesis.” Furthermore, the remote “origin” will automatically be added so that you can easily push your changes back to GitHub. You now have copies of your repository on your work computer, your GitHub account online, and your home computer. You can make changes, commit them on your home computer, and send those commits to the remote repository with git push, just as you did on your work computer.

Then the next day back at your work computer, you could update the code with the changes you made the previous evening using the command git pull.

$ git pull origin master

This pulls in all the commits that you had previously pushed to the GitHub remote repository from your home computer. In this workflow, you are essentially collaborating with yourself as you work from multiple computers. If you are working on a project with just one or two other collaborators, you could extend this workflow so that they could edit the code in the same way. You can do this by adding them as Collaborators on your repository (Settings -> Collaborators -> Add collaborator; see https://help.github.com/articles/adding-collaborators-to-a-personal-repository/). However, with projects with lots of contributors, GitHub provides a workflow for finer-grained control of the code development.

With the addition of a GitHub account and a few commands for sending and receiving code, you can now share your code with others, transfer your code across multiple machines, and set up simple collaborative workflows.

## Contribute to Other Projects

**Fig 4:**
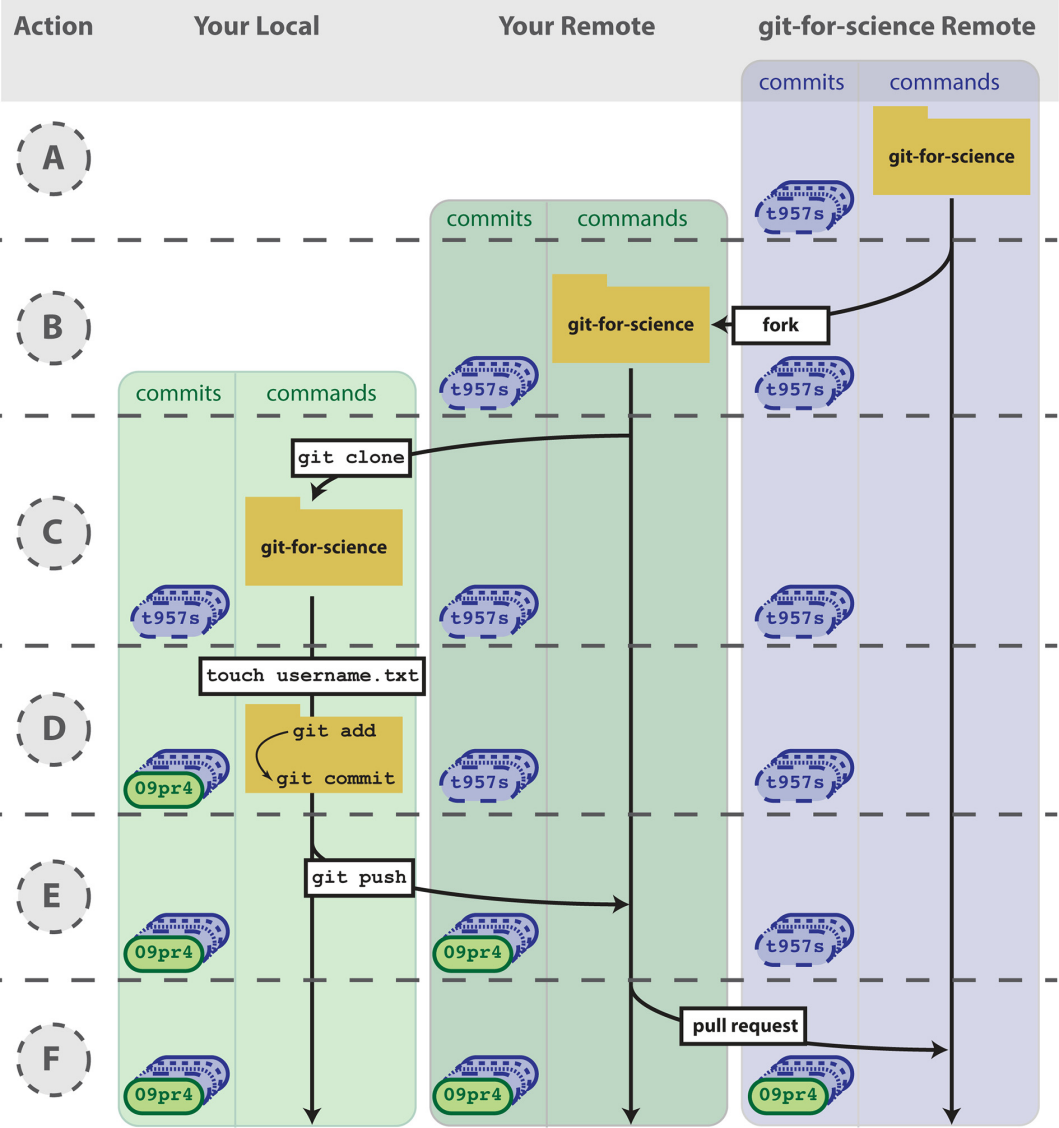
Contributing to open source projects. We would like you to add an empty file that is named after your GitHub username to the repo used to write this manuscript. (A) Using your internet browser, navigate to https://github.com/jdblischak/git-for-science. (B) Click on the “Fork” button to create a copy of this repo on GitHub under your username. (C) On your computer, type git clone https://github.com/username/git-for-science.git, which will create a copy of git-for-science on your local machine. (D) Navigate to the readers directory by typing cd git-for-science/readers/. Create an empty file that is titled with your GitHub username by typing touch username.txt. Commit that new file by adding it to the staging area (git add username.txt) and committing with a message (git commit -m "Add username to directory of readers."). Note that your commit identifier will be different than what is shown here. (E) You have committed your new file locally, and the next step is to push that new commit up to the git-for-science repo under your username on GitHub. To do so, type git push origin master. (F) To request to add your commits to the original git-for-science repo, issue a pull request from the git-for-science repo under your username on GitHub. Once your Pull Request is reviewed and accepted, you will be able to see the file you committed with your username in the original git-for-science repository.

Lots of scientific software is hosted online in Git repositories. Now that you know the basics of Git, you can directly contribute to developing the scientific software you use for your research (Fig 4). From a small contribution like fixing a typo in the documentation to a larger change such as fixing a bug, it is empowering to be able to improve the software used by yourself and many other scientists.

When contributing to a larger project with many contributors, you will not be able to push your changes with git push directly to the project’s remote repository. Instead, you will first need to create your own remote copy of the repository, which on GitHub is called a fork (Box 1). You can fork any repository on GitHub by clicking the button “Fork” on the top right of the page (see https://help.github.com/articles/fork-a-repo/).

Once you have a fork of a project’s repository, you can clone it to your computer and make changes just like a repository you created yourself. As an exercise, you will add a file to the repository that we used to write this paper. First, go to https://github.com/jdblischak/git-for-science and choose the “Fork” option to create a git-for-science repository under your GitHub account (Fig 4B). In order to make changes, download it to your computer with the command git clone from the directory you wish the repo to appear in (Fig 4C).

$ git clone https://github.com/username/git-for-science.git

Now that you have a local version, navigate to the subdirectory readers and create a text file named as your GitHub username (Fig 4D).

$ cd git-for-science/readers

$ touch username.txt

Add and commit this new file (Fig 4D), and then push the changes back to your remote repository on GitHub (Fig 4E).

$ git add username.txt

$ git commit -m "Add username to directory of readers."

$ git push origin master

Currently, the new file you created, readers/username.txt, only exists in your fork of git-for-science. To merge this file into the main repository, send a pull request using the GitHub interface (Pull request -> New pull request -> Create pull request; Fig 4F; see https://help.github.com/articles/using-pull-requests/). After the pull request is created, we can review your change and then merge it into the main repository. Although this process of forking a project’s repository and issuing a pull request seems like a lot of work to contribute changes, this workflow gives the owner of a project control over what changes get incorporated into the code. You can have others contribute to your projects using the same workflow.

The ability to use Git to contribute changes is very powerful because it allows you to improve the software that is used by many other scientists and also potentially shape the future direction of its development.

## Conclusion

Git, albeit complicated at first, is a powerful tool that can improve code development and documentation. Ultimately, the complexity of a VCS not only gives users a well-documented “undo” button for their analyses, but it also allows for collaboration and sharing of code on a massive scale. Furthermore, it does not need to be learned in its entirety to be useful. Instead, you can derive tangible benefits from adopting version control in stages. With a few commands (git init, git add, git commit), you can start tracking your code development and avoid a file system full of copied files (Fig 2). Adding a few additional commands (git push, git clone, git pull) and a GitHub account, you can share your code online, transfer your changes across machines, and collaborate in small groups (Fig 3). Lastly, by forking public repositories and sending pull requests, you can directly improve scientific software (Fig 4).

## Methods

We collaboratively wrote the article in LaTeX (http://www.latex-project.org/) using the online authoring platform Authorea (https://www.authorea.com). Furthermore, we tracked the development of the document using Git and GitHub. The Git repo is available at https://github.com/jdblischak/git-for-science, and the rendered LaTeX article is available at https://www.authorea.com/users/5990/articles/17489.

## Supporting Information

S1 Data process.sh. This Bash script downloads the ENCODE CTCF ChIP-seq data from multiple types of kidney samples and calls peaks. See https://github.com/jdblischak/git-for-science/tree/master/code for instructions on running it.
(SH) Click here for additional data file.

S2 Data clean.py. This Python script filters peaks with a fold change cutoff and merges peaks from the different kidney samples. See https://github.com/jdblischak/git-for-science/tree/master/code for instructions on running it.
(PY) Click here for additional data file.

S3 Data analyze.R. This R script creates diagnostic plots on the length of the peaks and their distribution across the genome. See https://github.com/jdblischak/git-for-science/tree/master/code for instructions on running it.
(R) Click here for additional data file.
